# Superior prognostic value of right ventricular free wall compared to global longitudinal strain in patients with repaired tetralogy of Fallot

**DOI:** 10.3389/fcvm.2022.996398

**Published:** 2022-09-26

**Authors:** Ying Gao, He Li, Lin He, Yanting Zhang, Wei Sun, Meng Li, Lang Gao, Yixia Lin, Mengmeng Ji, Qing Lv, Jing Wang, Li Zhang, Mingxing Xie, Yuman Li

**Affiliations:** ^1^Department of Ultrasound Medicine, Union Hospital, Tongji Medical College, Huazhong University of Science and Technology, Wuhan, China; ^2^Department of Ultrasound in Medicine, Shanghai Institute of Ultrasound in Medicine, Shanghai Jiao Tong University Affiliated Sixth People’s Hospital, Shanghai, China; ^3^Hubei Province Key Laboratory of Molecular Imaging, Wuhan, China; ^4^Clinical Research Center for Medical Imaging in Hubei Province, Wuhan, China

**Keywords:** tetralogy of Fallot, speckle tracking echocardiography, outcome, right ventricular function, strain

## Abstract

**Objectives:**

Both right ventricular free wall longitudinal strain (RVFWLS) and right ventricular global longitudinal strain (RVGLS) using two-dimensional speckle tracking echocardiography (2D-STE) has been demonstrated to predict adverse outcomes in patients with repaired tetralogy of Fallot (r-TOF). However, RVGLS may be affected by left ventricular (LV) function owing to the fact that the interventricular septum is also a part of the left ventricle. Therefore, the aim of our study was to compare the predictive value of RVFWLS with that of RVGLS in patients with r-TOF.

**Materials and methods:**

A total of 179 patients with r-TOF were included in this study. RVFWLS, RVGLS, and left ventricle global longitudinal strain (LVGLS) were evaluated by 2D-STE. The adverse clinical events were death or r-TOF-related rehospitalization. Prognostic performance was evaluated by C-statistic and Akaike information criterion (AIC).

**Results:**

Thirty-one patients developed poor outcomes during a median follow-up period of 2.8 years. Compared with patients without end-point events, those with end-point events had higher incidence of moderate/severe pulmonary regurgitation, larger right heart sizes, and lower RV fractional area change (RVFAC), RVFWLS, RVGLS, and LVGLS than those without. Multivariate Cox regression analysis revealed that RVFAC, RVFWLS, RVGLS, and LVGLS were predictive of poor outcomes in patients with r-TOF after adjustment for transannular patch and QRS duration. A Cox model using RVFWLS (C index = 0.876, AIC = 228) was found to predict unfavorable outcomes more accurately than a model with RVGLS (C index = 0.856, AIC = 243), RVFAC (C index = 0.811, AIC = 248), and LVGLS (C index = 0.830, AIC = 248).

**Conclusion:**

Although both RVGLS and RVFWLS are associated with adverse events, RVFWLS provides superior prognostic value than that of RVGLS in patients with r-TOF.

## Introduction

Tetralogy of Fallot (TOF) is the most common cyanotic congenital heart disease, with excellent survival into adulthood when surgical repair is performed in early childhood (1). However, the high prevalence of residual lesions and late complications, including right ventricular (RV) dysfunction, often require re-intervention (2). RV dysfunction confers a dismal long-term outcomes in patients with repaired tetralogy of Fallot (r-TOF), with reduced exercise tolerance and increased risk of life-threatening arrhythmia (LTA) and sudden cardiac death (SCD) (3–5). Therefore, early identification of RV dysfunction is crucial for patients with r-TOF at high risk of poor prognosis.

Currently, cardiac magnetic resonance (CMR) imaging remains the golden standard to evaluate RV function (6–8). However, it is more expensive and time-consuming than transthoracic echocardiography, hindering its clinical application for serial evaluation in patients with r-TOF. Echocardiography has been more widely used for post-operative follow-up in patients with r-TOF; whereas, currently available conventional echocardiographic RV parameters have limited accuracy (5). Speckle tracking echocardiography (STE) has been proposed as an more sensitive and accurate technique to assess RV function, which can quantify myocardial deformation from two-dimensional echo images without angle dependence (9, 10).

Both right ventricular global longitudinal strain (RVGLS) and right ventricular free wall longitudinal strain (RVFWLS) are obtained from STE. RVGLS refers to the average strain value of the RV free wall and the septal segments, whereas RVFWLS represents the average strain value of the RV free wall segments alone. Several studies demonstrated the prognostic value of RVGLS in r-TOF patients (10–13). In contrast, other studies focused on RVFWLS and showed that RVFWLS was associated with poor outcomes in patients with r-TOF (14, 15). As we know, interventricular septum consists of both the RV and left ventricular (LV) wall. Most investigators reckon that interventricular septum is mainly a part of the left ventricle. RVGLS might be influenced by LV function. Consequently, the aim of our study was to compare the predictive value of RVFWLS with that of RVGLS in patients with r-TOF.

## Materials and methods

### Study population

This retrospective study included clinically stable patients with primary correction for TOF from January 1, 2000 to December 31, 2015. Exclusion criteria were pulmonary atresia, absent pulmonary valve, atrioventricular canal anomaly; palliative correction or staged correction; patients with poor image quality. For patients who had more than one echocardiographic examination during this period, only the first examination was used. This study was approved by the Ethics Committee of Tongji Medical College, Huazhong University of Science and Technology.

### Clinical data

Demographics, medical history, QRS duration, TOF anatomical type, age at repair, surgery method (primary/staged), surgical approach (*via* right ventricle/right atrium/pulmonary artery), with/without transannular patch (TAP), cardiopulmonary bypass time (CPBT), aortic cross-clamping time (ACCT), and intensive care unit (ICU) stay were collected from medical records.

### Conventional echocardiography

All echocardiographic examinations were performed using Philips echocardiographic systems (IE 33 and EPIQ 5; S5-1, X5-1 transducer; Philips Healthcare, Andover, MA, USA). Post-operative echocardiographic images were obtained from the ultrasound PCAS system. All measurements were acquired according to the guidelines of the American Society of Echocardiography (16). Diastolic diameter of right ventricular outflow tract (RVOT), right ventricular end-diastolic area (RVEDA), and right atrium area (RAA) were evaluated in the apical 4-chamber view. Peak systolic (S′) velocity of tricuspid annulus was measured by pulsed TDI, placing the sample volume at the level of tricuspid lateral annulus from the apical 4-chamber view. RV fractional area change (RVFAC) were calculated as (RV end-diastolic area–RV end-systolic area)/end-diastolic area × 100%. Tricuspid annular plane systolic excursion (TAPSE) and mitral annular plane systolic excursion (MAPSE) were measured by the M-mode in the apical 4-chamber view. LV ejection fraction (LVEF) was determined by the biplane Simpson method.

### Two-dimensional speckle tracking echocardiography analysis

**FIGURE 1:**
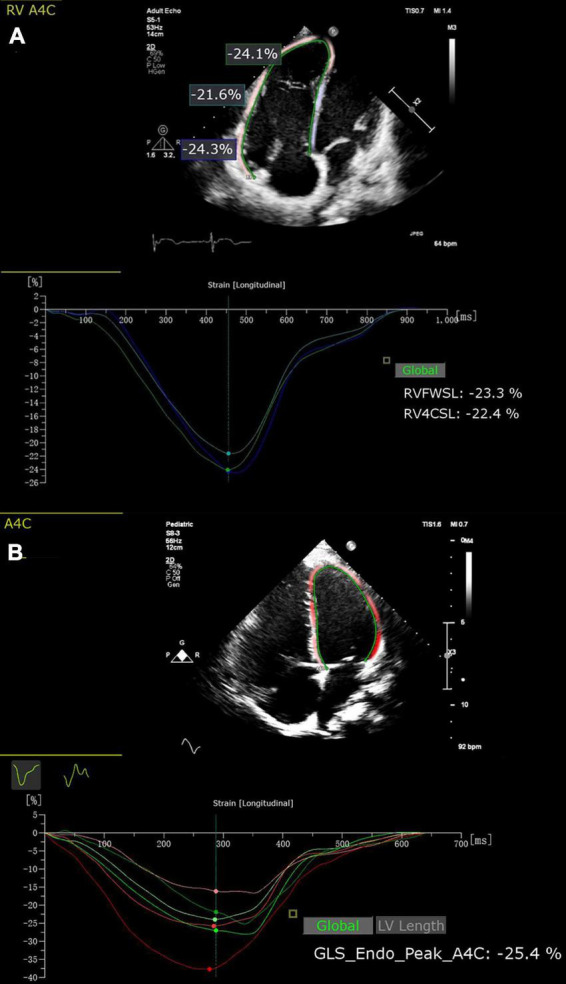
Two-dimensional speckle tracking echocardiography (2D-STE) offline analysis for RVFWLS **(A)**, RVGLS **(A)** and LVGLS **(B)**. RVFWSL, right ventricular free wall longitudinal strain; RV4CSL, right ventricular global longitudinal strain.

Two-dimensional speckle tracking echocardiography (2D-STE) analysis for right ventricle and left ventricle was performed with commercially available software (AutoStrain; Tomtec Imaging Systems, Unterschleißheim, Germany). RVFWLS and RVGLS were evaluated in the apical four-chamber view. For each patient, after manual registration of an apical four-chamber view, the RV endocardial border at end-diastole was automatically determined. We have manually corrected RV endocardial borders if necessary. Right ventricle was automatically segmented into six segments (basal, middle, and apical segments of both the RV free wall and the interventricular septum). Finally, the longitudinal strain curves for each RV segment were automatically generated by the software. RVFWLS was calculated as the average value of the three segments of the RV free wall. RVGLS was calculated by averaging strain values of the RV six segments. Left ventricle global longitudinal strain (LVGLS) was assessed in the apical four-chamber view using the same methodology (Figure 1).

### Definition and assessment of events

The adverse clinical events were death or r-TOF-related rehospitalization, including pulmonary valve replacement (PVR) due to pulmonary regurgitation, permanent pacemaker implantation owing to arrhythmia, reoperation for residual obstruction or residual VSD, rehospitalization due to cardiac dysfunction (RVFAC < 35% or LVEF < 50% with clinical symptoms of heart failure). The follow-up period was up to December 30, 2020.

### Statistical analysis

Continuous variables are expressed as mean ± SD for normally distributed data or median (interquartile range, IQR) for non-normally distributed data. Categorical variables are presented as frequency (percentage). Continuous variables were compared using analysis of variance (ANOVA) for normally distributed data, and the Mann–Whitney *U*-test or Kruskal–Wallis *H*-test for non-normally distributed data. Categorical variables were compared using the chi-square test or the Fisher exact test.

Estimations of the predictor of adverse events were performed using univariate and multivariate Cox regression models. All potential predictors of adverse outcome were entered into univariate analyses. Variables with *P* < 0.001 at univariate analysis were entered into multivariate Cox regression models. For multivariable analysis, a separate model including clinical variables and one of STE parameters was used to determine the independent predictors of poor outcome. To investigate the model performance, we compared their C-index and Akaike information criterion (AIC). Receiver operator characteristic (ROC) analysis was performed to examine the sensitivity and specificity of prognosis parameters for adverse events, and determine the best cutoff value (maximum Youden index) for predicting future events. Kaplan-Meier curves were used to examine cumulative event rates, and compared their differences between the two subgroups using the log rank test. Statistical analyses were performed using the SPSS version 26.0 (SPSS Inc., Chicago, IL, USA), R-language 4.1.1 and the GraphPad Prism 8. A two-sided value of *P* < 0.05 was considered as statistically significant.

We randomly selected 30 patients and evaluated the intraobserver and interobserver variability of LVGLS, RVGLS and RVFWLS using intra-class correlation coefficient (ICC) and coefficient of variation. To assess the intraobserver variability, the data were remeasured independently after 1 month by the same operator. To evaluate the interobserver variability, the data were reanalyzed by the second operator who was blinded to the first measurement.

## Results

### Patient characteristics

There were 356 patients with TOF who underwent primary repair in our hospital from 2000 to 2015. Following repair, 318 were discharged alive. While 139 who did not conform to the inclusion criteria were excluded, of which 93 were lost to follow-up and 46 had poor image quality. A total of 179 patients with r-TOF who underwent at least one post-operative echocardiography examinations (selecting the first examination) and meet the quality criteria for strain analysis before developing to the endpoint, were included in our study. The median age was 8 years (IQR, 5–18 years). 119 patients (66.5%) were male. All patients were divided into three groups according to the tertiles of RVFWLS (lower, middle, and upper tertiles). The lower, middle, and upper tertiles of RVFWLS were −4.3∼−17.7%, −17.8∼−21.1%, and −21.2∼−30.0% respectively.

The baseline clinical and echocardiographic characteristics of patients stratified by the tertiles of RVFWLS are described in Table 1. Compared with patients in the highest RVFWLS tertile, those in the lowest tertile had prolonged QRS duration. There were no significant differences in the gender, age, heart rate at examination, and the remaining surgical characteristics (including the age at repair, VSD repair approach *via* RV, TAP, concomitant procedures, ACCT, CPBT, ICU stay) in patients among the low, moderate, and high tertiles. Regarding echocardiographic findings, patients in the lowest RVFWLS tertile exhibited higher incidence of moderate/severe pulmonary regurgitation, and lower RVFAC, S′, MAPSE, LVGLS, RVGLS, and RVFWLS than those in the highest tertile. The incidence of residual VSD and RVOT obstruction, right heart sizes, TAPSE and LVEF were not different among the tertiles.

**TABLE 1:** Post-operative baseline characteristics of patients with r-TOF according to tertiles of RVFWLS.

	All patients −4.3%∼−30.0% (*n* = 179)	RVFWLS			*P*-value
		
		Lower tertile −4.3%∼−17.7% (*n* = 59)	Middle tertile −17.8%∼−21.1% (*n* = 60)	Upper tertile −21.2%∼−30.0% (*n* = 60)	
**Demographic data**					
Male, *n* (%)	119 (66.5)	40 (67.8)	40 (66.7)	39 (65.0)	0.948
Age, years	8 [5, 18]	8 [5, 26]	8 [5, 15]	8 [5, 11]	0.859
Heart rate, bpm	78 [71, 91]	78 [68, 90]	79 [72, 92]	78 [72, 92]	0.399
QRS duration, ms	118.8 ± 17.5	125.7 ± 17.8	114.7 ± 16.3	116.2 ± 16.7	0.001
**Surgical characteristics**					
Age at repair, years	1.1 [0.6, 9.4]	1.2 [0.6, 18.0]	1.0 [0.6, 3.6]	1.3 [0.8, 5.2]	0.625
Era 2000–2005/2005–2010/2010–2015	12/76/91	2/27/30	7/15/38	3/34/23	0.005
TAP, *n* (%)	88 (49.2)	34 (57.6)	31 (51.7)	23 (38.3)	0.097
VSD repair approach *via* RV, *n* (%)	24 (13.4)	9 (15.3)	9 (15.0)	6 (10.0)	0.636
Concomitant procedures, *n* (%)	64 (35.8)	18 (30.5)	19 (31.7)	27 (45.0)	0.185
CPBT, min	95 [80, 119]	97 [79, 132]	93 [82, 112]	95 [77, 120]	0.786
ACCT, min	50 [38, 64]	53 [40, 69]	46 [36, 63]	53 [36, 64]	0.424
ICU stay, day	5 [4, 7]	5 [4, 6]	6 [4, 7]	6 [4, 7]	0.674
**Post-operative echocardiogram data**					
Residual obstruction, n (%)	12 (6.7)	7 (11.9)	1 (1.7)	4 (6.7)	0.072
Residual VSD, *n* (%)	41 (22.9)	16 (27.1)	17 (28.3)	8 (13.3)	0.095
Moderate/severe PR, *n* (%)	113 (63.1)	42 (71.2)	41 (68.3)	30 (50.0)	0.034
LVEF, %	63.3 ± 6.8	63.4 ± 7.3	62.4 ± 7.8	64.2 ± 4.9	0.212
MAPSE, mm	12.1 ± 3.7	11.0 ± 3.4	12.2 ± 4.0	13.3 ± 3.5	0.010
RVOT, mm	25.2 ± 7.9	26.8 ± 9.0	25.1 ± 6.6	23.6 ± 7.4	0.076
RVEDA, cm^2^	28.9 ± 11.4	31.4 ± 13.0	28.2 ± 10.9	27.2 ± 9.8	0.127
RAA, cm^2^	15.2 ± 6.4	16.5 ± 7.3	14.8 ± 6.6	14.2 ± 5.0	0.138
RVFAC, %	37.3 ± 5.9	34.8 ± 6.8	37.2 ± 5.4	40.0 ± 4.2	<0.001
TAPSE, mm	13.8 ± 3.8	13.2 ± 4.3	13.7 ± 3.4	14.7 ± 3.5	0.057
S’(TV), cm/s	6.3 ± 2.1	5.2 ± 1.6	5.9 ± 1.6	7.6 ± 2.2	<0.001
**2D-STE data**					
LVGLS, %	−19.2 ± 4.0	−16.2 ± 3.4	−19.4 ± 3.6	−21.8 ± 2.6	<0.001
RVFWLS, %	−19.5 ± 4.4	−14.8 ± 2.6	−19.5 ± 1.0	−24.3 ± 2.4	<0.001
RVGLS, %	−18.8 ± 3.7	−15.2 ± 2.9	−19.0 ± 1.7	−22.2 ± 2.1	<0.001
**Event, *n* (%)**	31 (17.3)	27 (45.8)	4 (6.7)	0 (0.0)	<0.001

Data are n (%), Median [IQR] or mean ± SD.

*P*-values comparing patients with r-TOF in different groups are from χ^2^-test, Fisher’s exact test, ANOVA or Mann–Whitney U-test. P < 0.05 was considered statistically significant. IQR, interquartile range; TAP, transannular patch; VSD, ventricular septal defect; CPBT, cardiopulmonary bypass time; ACCT, aortic cross-clamping time; ICU, intensive care unit; PR, pulmonary regurgitation; LVEF, left ventricular ejection fraction; MAPSE, mitral annular plane systolic excursion; RVOT, right ventricular outflow tract; RVEDA, right ventricular end-diastolic area; RAA, right atrium area; RVFAC, right ventricular fractional area change; TAPSE, tricuspid annular plane systolic excursion; S’(TV), peak systolic velocity of tricuspid annulus; LVGLS, left ventricular global longitudinal strain; RVFWLS, right ventricular free wall longitudinal strain; RVGLS, right ventricular global longitudinal strain.

During a median follow-up duration of 2.8 years (IQR, 1.4–10.0 years), 31 patients with r-TOF (17.3%) reached the predefined endpoint of adverse events. Eight patients underwent PVR due to severe pulmonary regurgitation, one patient underwent permanent pacemaker implantation because of severe ventricular arrhythmia, Two patients underwent recanalization for residual obstruction, one patient underwent repair for residual VSD, and 19 patients were rehospitalized due to cardiac dysfunction.

Table 2 shows clinical and echocardiographic characteristics of patients with or without events. Compared with patients with adverse events, those without adverse events were more likely to have prolonged QRS duration, and higher incidence of TAP, VSD repair approach *via* RV and residual obstruction. Regarding echocardiographic data, patients with adverse events displayed higher incidence of moderate/severe pulmonary regurgitation, larger right heart sizes, and lower MAPSE, RVFAC, TAPSE, S′, RVFWLS, RVGLS, and LVGLS than those without. The incidence of residual VSD and LVEF did not differ between two subgroups.

**TABLE 2:** Post-operative baseline characteristics of r-TOF patient with and without adverse event.

	All patients (*n* = 179)	Without event (*n* = 148)	With event (*n* = 31)	*P*-value
**Demographic data**				
Male, *n* (%)	119 (66.5)	98 (66.2)	21 (67.7)	1
Age, years	8 [5, 18]	8 [5, 13]	19 [4, 30]	0.137
Heart rate, bpm	78 [71, 91]	78 [71, 92]	78 [71, 86]	0.806
QRS duration, ms	118.8 ± 17.5	115.2 ± 16.1	136.3 ± 13.3	<0.001
**Surgical characteristics**				
Age at repair, years	1.1 [0.6, 9.4]	1.1 [0.6, 4.0]	13.7 [0.6, 25.1]	0.054
Era 2000–2005/2005–2010/2010–2015	12/76/91	8/65/75	4/11/16	0.256
TAP, *n* (%)	88 (49.2)	62 (41.9)	26 (83.9)	<0.001
VSD repair approach *via* RV, *n* (%)	24 (13.4)	16 (10.8)	8 (25.8)	0.039
Concomitant procedures, *n* (%)	64 (35.8)	55 (37.2)	9 (29.0)	0.419
CPBT, min	95 [80, 119]	95 [80, 116]	93 [78, 145]	0.744
ACCT, min	50 [38, 64]	50 [37, 63]	49 [39, 85]	0.207
ICU stay, day	28 [20, 37]	6 [4, 7]	5 [4, 6]	0.308
**Post-operative echocardiogram data**				
Residual obstruction, *n* (%)	12 (6.7)	6 (4.1)	6 (19.4)	0.007
Residual VSD, *n* (%)	41 (22.9)	31 (20.9)	10 (32.3)	0.238
Moderate/severe PR, *n* (%)	113 (63.1)	86 (58.1)	27 (87.1)	0.002
LVEF, %	63.3 ± 6.8	63.4 ± 6.5	62.9 ± 8.1	0.930
MAPSE, mm	12.1 ± 3.7	12.5 ± 3.9	10.8 ± 2.6	0.037
RVOT, mm	25.2 ± 7.9	24.4 ± 7.6	28.5 ± 8.3	0.015
RVEDA, cm^2^	28.9 ± 11.4	28.0 ± 10.9	33.3 ± 12.8	0.021
RAA, cm^2^	15.2 ± 6.4	14.6 ± 6.0	17.4 ± 7.1	0.044
RVFAC,%	37.3 ± 5.9	38.3 ± 5.1	32.6 ± 7.2	<0.001
TAPSE, mm	13.8 ± 3.8	14.1 ± 3.9	12.4 ± 2.7	0.028
S’(TV), cm/s	6.3 ± 2.1	6.6 ± 2.1	5.0 ± 1.8	0.003
**2D-STE data**				
LVGLS, %	−19.2 ± 4.0	−19.8 ± 3.7	−16.0 ± 3.7	<0.001
RVFWLS, %	−19.5 ± 4.4	−20.5 ± 3.9	−14.8 ± 3.3	<0.001
RVGLS, %	−18.6 ± 3.8	−19.5 ± 3.3	−14.4 ± 3.4	<0.001

Data are n (%), Median [IQR] or mean ± SD.

*P*-values comparing patients with r-TOF in different groups are from χ^2^-test, Fisher’s exact test, ANOVA or Mann–Whitney U-test. P < 0.05 was considered statistically significant. IQR, interquartile range. TAP, transannular patch; VSD, ventricular septal defect; CPBT, cardiopulmonary bypass time; ACCT, aortic cross-clamping time; ICU, intensive care unit; PR, pulmonary regurgitation; LVEF, left ventricular ejection fraction; MAPSE, mitral annular plane systolic excursion; RVOT, right ventricular outflow tract; RVEDA, right ventricular end-diastolic area; RAA, right atrium area; RVFAC, right ventricular fractional area change; TAPSE, tricuspid annular plane systolic excursion; S’(TV), peak systolic velocity of tricuspid annulus; LVGLS, left ventricular global longitudinal strain; RVFWLS, right ventricular free wall longitudinal strain; RVGLS, right ventricular global longitudinal strain.

### Incremental predictive value of right ventricular free wall longitudinal strain over right ventricular global longitudinal strain

Univariable Cox regression analysis showed that QRS duration (HR: 1.043, 95% CI: 1.025∼1.061; *P* < 0.001), TAP (HR: 6.218, 95% CI: 2.383∼16.222; *P* < 0.001), RVFAC (HR: 0.869, 95% CI: 0.824∼0.917; *P* < 0.001), S′ (HR: 0.662, 95% CI: 0.475∼0.923; *P* = 0.015), RVFWLS (HR: 1.258, 95% CI: 1.173∼1.348; *P* < 0.001), RVGLS (HR: 1.241, 95% CI: 1.150∼1.339; *P* < 0.001), and LVGLS (HR: 1.197, 95% CI: 1.103∼1.299; *P* < 0.001) were associated with adverse events in patients with r-TOF (Table 3).

**TABLE 3:** Predictors of adverse event in patients with r-TOF by univariate cox regression analysis.

Variables	HR (95% CI)	*P*-value
TAP	6.218 (2.383, 16.222)	<0.001
QRS duration, ms	1.043 (1.025, 1.061)	<0.001
VSD repair approach *via* RV	2.910 (1.295, 6.538)	0.010
Moderate/severe PR	5.356 (1.866, 15.377)	0.002
MAPSE, mm	0.900 (0.812, 0.998)	0.045
RVOT, mm	1.057 (1.013, 1.103)	0.011
RVEDA, cm^2^	1.049 (1.021, 1.078)	0.001
RAA, cm^2^	1.090 (1.036, 1.147)	0.001
RVFAC, %	0.869 (0.824, 0.917)	<0.001
TAPSE, mm	0.936 (0.848, 1.033)	0.188
S’(TV), cm/s	0.662 (0.475, 0.923)	0.015
LVGLS, %	1.197 (1.103, 1.299)	<0.001
RVFWLS, %	1.258 (1.173,1.348)	<0.001
RVGLS, %	1.241 (1.150,1.339)	<0.001

*P* < 0.05 was considered statistically significant.

AIC, Akaike information criterion; BIC, Bayesian information criterion; C-index, concordance index; CI, confidence interval; HR, hazard ratio. TAP, transannular patch; VSD, ventricular septal defect; PR, pulmonary regurgitation; MAPSE, mitral annular plane systolic excursion; RVOT, right ventricular outflow tract; RVEDA, right ventricular end-diastolic area; RAA, right atrium area; RVFAC, right ventricular fractional area change; TAPSE, tricuspid annular plane systolic excursion; S’(TV), peak systolic velocity of tricuspid annulus; LVGLS, left ventricular global longitudinal strain; RVFWLS, right ventricular free wall longitudinal strain; RVGLS, right ventricular global longitudinal strain.

The aforementioned significant predictors (*P* < 0.001), including QRS duration, TAP, RVFAC, RVFWLS, RVGLS, and LVGLS were incorporated into the multivariate Cox regression analysis (Table 4). To avoid the problems of overfitting and collinearity, separate multivariable models including QRS duration, TAP and one of cardiac function parameters (RVFAC, RVFWLS, RVGLS, and LVGLS) was used to determine the independent predictors of poor outcome. A model with RVFWLS (C index = 0.876, AIC = 228) was found to be the best in predicting adverse clinical events compared with the base model (adjusted for QRS duration and TAP, C index = 0.795, AIC = 254), a model with LVGLS (C index = 0.830, AIC = 248), RVFAC (C index = 0.811, AIC = 248), and RVGLS (C index = 0.856, AIC = 243).

**TABLE 4:** Predictors of adverse event in patients with r-TOF by multivariate cox regression analysis.

Variables	M0		M1		M2		M3		M4	
					
	HR (95% CI)	*P*-value	HR (95% CI)	*P*-value	HR (95% CI)	*P*-value	HR (95% CI)	*P*-value	HR (95% CI)	*P*-value
TAP	4.882 (1.844, 12.925)	0.001	3.983 (1.467, 10.815)	0.007	4.608 (1.734, 12.245)	0.002	3.754 (1.364, 10.335)	0.011	3.415 (1.247, 9.348)	0.017
QRS duration, ms	1.035 (1.018, 1.053)	<0.001	1.027 (1.009, 1.045)	0.003	1.024 (1.005,1.043)	0.014	1.023 (1.004, 1.043)	0.016	1.024 (1.005, 1.044)	0.014
LVGLS, %	–	–	1.131 (1.035, 1.236)	0.007	–	–	–	–	–	–
RVFAC, %	–	–	–	–	0.916 (0.864, 0.971)	0.003	–	–	–	–
RVFWLS, %	–	–	–	–	–	–	1.263 (1.156, 1.379)	<0.001	–	–
RVGLS, %	–	–	–	–	–	–	–	–	1.241 (1.137, 1.354)	<0.001
C-index	0.795	–	0.83	–	0.811	–	0.876	–	0.856	–
AIC	254	–	248	–	248	–	228	–	243	–

M0: TAP + QRS duration.

M1: TAP + QRS duration + LVGLS.

M2: TAP + QRS duration + RVFAC.

M3: TAP + QRS duration + RVFWLS.

M4: TAP + QRS duration + RVGLS.

AIC, Akaike information criterion; C-index, concordance index; CI, confidence interval; HR, hazard ratio. *P* < 0.05 was considered statistically significant.

**FIGURE 2:**
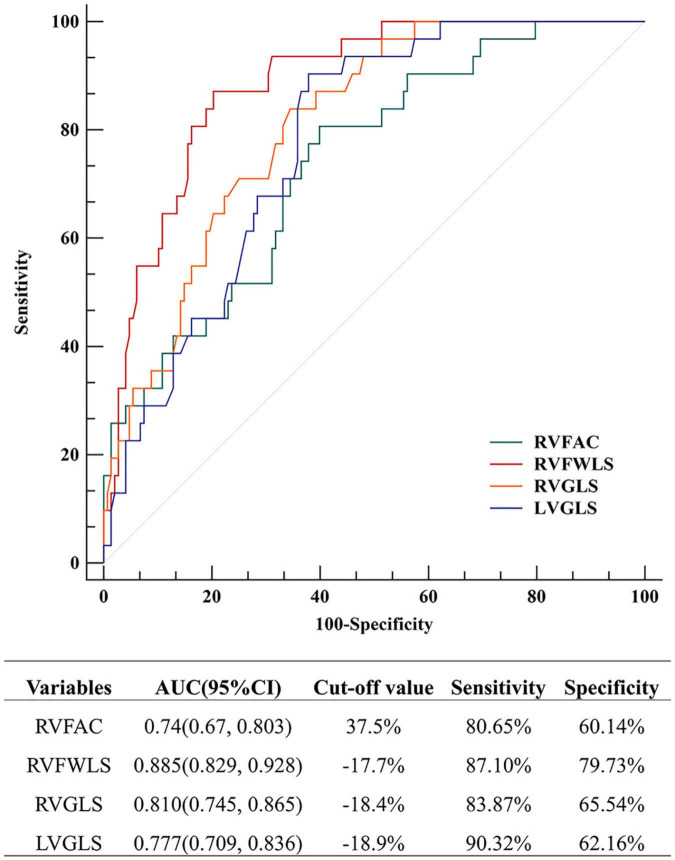
Receiver operator characteristic (ROC) analysis for prediction of adverse events in patients with r-TOF; ROC, receiver operating characteristic.

Receiver operator characteristic analysis was used to assess the predictive values of the aforementioned predictors (RVFAC, RVFWLS, RVGLS, and LVGLS) for adverse events in patients with r-TOF (Figure 2). The results showed that the area under curves (AUC) of RVFWLS (0.885) was greater than that of RVGLS (0.810, *P* = 0.015), LVGLS (0.777, *P* = 0.021), and RVFAC (0.740, *P* = 0.010). The best cutoff values of RVFWLS and RVGLS for detecting adverse outcomes were −17.7 and −18.4%, respectively.

**FIGURE 3:**
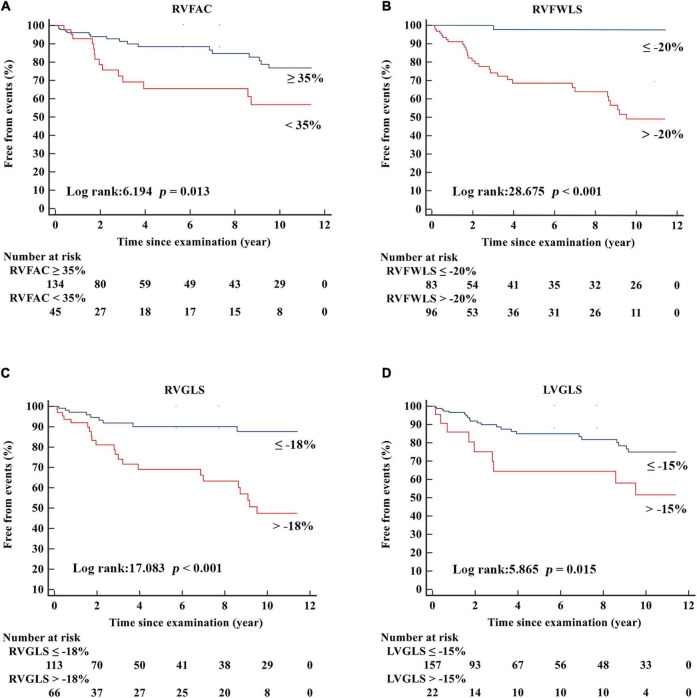
Kaplan–Meier freedom from events curves according to **(A)** RVFAC, **(B)** RVFWLS, **(C)** RVGLS, **(D)** LVGLS in patients with r-TOF.

The Kaplan-Meier estimates of adverse events are presented in Figure 3. In our study, we used the following cutoff values: 35% for RVFAC, −20% for RVFWLS (16), −18% for RV strain (16–18), and −15% for LVGLS (19). Patients with RVFAC < 35%, RVFWLS > −20%, RVGLS > −18%, and LVGLS > −15% had a higher risk of adverse events in patients with r-TOF.

### Reproducibility of speckle tracking echocardiography measurements

The intraobserver and interobserver variability for STE measurements are shown in Table 5. RVFWLS, RVGLS, and LVGLS presented with good reproducibility, as reflected by high ICCs.

**TABLE 5:** Reproducibility of STE indices.

Variables	ICC (95% CI)	CV
**Intra-observers (*n* = 30)**		
LVGLS, %	0.85 (0.70–0.92)	0.10
RVFWLS, %	0.84 (0.68–0.92)	0.13
RVGLS, %	0.88 (0.77–0.94)	0.10
**Inter-observers (*n* = 30)**		
LVGLS, %	0.84 (0.68–0.92)	0.11
RVFWLS, %	0.86 (0.72–0.93)	0.14
RVGLS, %	0.85 (0.71–0.93)	0.13

Data are expressed as mean ± SD.

LOA, limits of agreement; ICC, intra-class correlation coefficient; CV, coefficient of variation. LVGLS, left ventricular global longitudinal strain; RVFWLS, right ventricular free wall longitudinal strain; RVGLS, right ventricular global longitudinal strain.

## Discussion

To the best of our knowledge, this may be the first study to directly compare RVFWLS RVGLS, LVGLS, and conventional echocardiographic parameters and investigate their prognostic value in patients with r-TOF. The major findings of our study were: (1) compared with r-TOF patients without adverse events, those with adverse events displayed decreased RVFWLS, RVGLS, and LVGLS. (2) RVFWLS, RVGLS, and LVGLS remained significantly associated with the clinical outcomes after adjustment for TAP, QRS duration. (3) More importantly, our results demonstrated that RVFWLS better predicted adverse events than RVGLS or LVGLS in patients with r-TOF.

### Prognostic value of myocardial deformation in patients with repaired tetralogy of Fallot

In our study, a univariate Cox regression analysis demonstrated that LVGLS was associated with the adverse events, and LVGLS is still independently predictive of adverse clinical events after adjusting for TAP and QRS. These findings are in accordance with previous studies which have found that LVGLS can predict adverse outcomes, using CMR or echocardiography. Orwat et al. (11) showed that LVGLS based on CMR images was a predictor of SCD or ventricular tachycardia in patients with r-TOF. On echocardiography, LVGLS has been found to be associated with LTA/SCD by the research of Diller et al. (19). However, in the study of van Grootel et al. (15), LVGLS was not independently associated with increased risk of death or heart failure in the ridge regression analysis in which RVFWLS was also involved. van Grootel et al. (15) revealed that LVGLS may not be used to evaluate prognosis solely and RV longitudinal strain should be preferred.

Our study revealed that both RVFWLS and RVGLS were predictive of an increased risk of unfavorable clinical events in a univariate Cox regression analysis. More importantly, RVFWLS and RVGLS remained independently associated with adverse events after adjustment for clinical factors. Indeed, our study also showed that patients with end-point events had decreased RVFWLS and RVGLS compared with those without end-point events. These results are consistent with previous studies, which demonstrated the prognostic value of RVFWLS or RVGLS (assessed by CMR or 2D-STE) in patients with r-TOF (10–15).

In addition, our study also demonstrated that both RVFWLS and RVGLS may offer additional prognostic significance over RVFAC in patients with r-TOF. Diller et al. (19) revealed that RVFAC was associated with the end point of SCD/LTA. However, they did not compare the predictive implication of RV longitudinal strain with RVFAC. In contrast, van Grootel et al. (15) found that RVFAC could not predict cardiovascular events in patients with r-TOF. This may be the fact that RV longitudinal strain can easily identify the maximal and minimal values of myocardium deformation by tracking the myocardium throughout the cardiac cycle. However, RVFAC measurement merely relies on end-diastole and end-systole frames. Therefore, RV longitudinal strain may be a better prognostic marker in patients with r-TOF than RVFAC.

### Incremental predictive value of right ventricular free wall longitudinal strain over right ventricular global longitudinal strain in patients with repaired tetralogy of Fallot

Right ventricular longitudinal strain assessed by 2D-STE has been reported to be a powerful and independent predictor in various clinical settings, providing additional predictive value over conventional echocardiographic parameters (20–24). However, in patients with r-TOF, some researchers measured RVGLS (which include both RV free wall and interventricular septum), whereas other investigators focused on the analysis of RVFWLS. Since RVGLS might be influenced by LV function, a direct comparison between RVFWLS and RVGLS for predicting adverse clinical outcomes is clinically significant. Till now, there is no study to investigate which RV strain parameter better predict clinical events in patients with r-TOF.

Currently, several studies performed comparisons of RVGLS and RVFWS (25–27). Moreover, their results are discordant. In a mixed cohort of “left heart disease” of various etiologies, García-Martín et al. (25) compared the prognostic role of RVFWLS with RVGLS; they demonstrated that RVGLS better predicted adverse events. Similarly, in a series of patients undergoing cardiac resynchronization therapy, Nagy et al. (26) measured RVFWLS and RVGLS; they found that RVGLS was predictive of mortality, whereas RVFWLS displayed a tendency for mortality prediction. In contrast, Carluccio et al. (27) compared the prognostic value of RVFWLS and RVGLS in patients with heart failure and reduced ejection fraction. They revealed that RVFWLS remained independently associated with outcomes after adjusting for LVGLS and other echocardiographic predictors, whereas RVGLS was no longer predictive of clinical outcomes. Likewise, Costa Junior et al. (28) compared STE-determined RV longitudinal strain with CMR-derived RV function parameters, and found that RVFWLS was the strongest related to RVEF assessed by CMR and the most powerful predictor of outcomes in patients with pulmonary hypertension.

Our study for the first time showed that RVFWLS is more powerful than RVGLS in predicting adverse events in patients with r-TOF by comparing the C index and AIC of the multivariate COX regression model. This may be since that RVGLS includes the ventricular septum wall, which mediates the interactions between left and right ventricle. Therefore, RVGLS is more likely to be affected by the longitudinal function of the left ventricle. We offer the first evidence that RVFWLS was superior to RVGLS for predicting adverse outcomes in patients with r-TOF. In summary, the present study reinforces and expands the previous studies by indicating the prognostic utility of the RV longitudinal strain in the risk stratification of patients with r-TOF. Our study not only verifies the prognostic value of both RVFWLS and RVGLS in patients with r-TOF but also demonstrates the incremental prognostic value of RVFWLS over RVGLS. As mentioned above, previous studies have shown that RV dysfunction confers a dismal long-term outcome in patients with r-TOF, with reduced exercise tolerance and increased risk of LTA and SCD. Considering that RVFWLS reflects the RVEF-CMR (28) and RVGLS might be influenced by LV function, it is not surprising that RVFWLS is the most powerful predictor of adverse endpoints in the present study. Our study revealed the imperative clinical implication of RVFWLS, since it can be easily acquired from echocardiography. Our results indicate that assessment of RV function using traditional echocardiographic parameters should be complemented by RV longitudinal strain analysis to detect patients who are at higher risk for adverse events.

### Limitations

This was a single-center retrospective study, which may cause inclusion bias. Because of the limited number of adverse events and relatively short follow-up, we defined a composite endpoint. Future studies with larger sample sizes and longer follow-up are required to further verify our findings. In addition, STE analysis relies on high-quality echocardiographic images, the additional prognostic value of RVFWLS over RVGLS in r-TOF patients with poor image quality cannot be determined. Another limitation of our study is that strain measurements display vendor variation. Thus, our findings might not be extrapolated to other vendors. Finally, because CMR is not included in the routine follow-up, this study lacks CMR data for comparison.

## Conclusion

Our study strengthens and expands previous studies by demonstrating that both RVFWLS and RVGLS provide additional predictive value over clinical parameters, LVGLS and conventional echocardiographic indices in patients with r-TOF. Moreover, RVFWLS better predicts clinical outcomes than RVGLS in patients with r-TOF. Therefore, it is recommended to measure RVFWLS for risk stratification in patients with r-TOF during routine follow-up.

## Data availability statement

The raw data supporting the conclusions of this article will be made available by the authors, without undue reservation.

## Ethics statement

This study was approved by the Ethics Committee of Tongji Medical College, Huazhong University of Science and Technology. Written informed consent was not required for this study in accordance with the local legislation and institutional requirements.

## Author contributions

YL, MX, and LZ designed the study and were guarantor of the manuscript. YG, HL, and LH prepared the draft and finalized the manuscript. YG helped with data analysis and interpretation of the results. YZ, WS, ML, LG, YL, and MJ were involved in clinical data collection and arrangement. QL and JW participated in ultrasound imaging acquisition in isolation wards. All authors contributed to the article and approved the submitted version.
